# Effects of Aquatic Exercise in Post-exercise Hypotension: A Systematic Review and Meta-Analysis

**DOI:** 10.3389/fphys.2022.834812

**Published:** 2022-01-31

**Authors:** Cristina Oliveira Trindade, Emerson Cruz Oliveira, Daniel Barbosa Coelho, Juliano Casonatto, Lenice Kappes Becker

**Affiliations:** ^1^Postgraduate Program in Health and Nutrition/PPGSN, Federal University of Ouro Preto, Ouro Preto, Brazil; ^2^Physical Education Department, Physical Education School, Federal University of Ouro Preto, Ouro Preto, Brazil; ^3^Research Group in Physiology and Physical Activity, University of Northern Paraná, Londrina, Brazil

**Keywords:** post-exercise hypotension, aquatic exercise, water-based exercise, exercise, ambulatory blood pressure, systematic review and meta-analysis

## Abstract

**Background:**

Post-exercise hypotension (PEH) can be an important non-pharmacological strategy in the treatment of arterial hypertension. Both aerobic and resistance exercises produce PEH, but it is not clear if the exercise environment can lead to a higher PEH.

**Objective:**

This meta-analysis investigated whether a session of aquatic exercise (AE) induces PEH in comparison with control conditions such as land exercise (LE) or rest in hypertensive subjects.

**Methods:**

The present systematic review and meta-analysis was conducted using the following electronic databases: PubMed, Google Scholar, and EMBASE. Ambulatory blood pressure measurements made in randomized clinical trials were pooled to compare PEH induced by AE with LE and rest conditions in hypertensive subjects.

**Results:**

Data from four trials were included, which comprised 127 participants (94 women and 33 men). A 24-h analysis did not detect significant differences between AE and LE or rest for either systolic blood pressure (SBP) or diastolic blood pressure (DBP). Monitoring during the night showed that AE induced significant PEH in comparison with LE for SBP [−8.6 (−15.0 to −1.5) mmHg (*p* = 0.01)]. For DBP, the AE had pronounced PEH during the night in comparison with LE [−3.7 (−4.7 to −2.8) mmHg, *p* = 0.000] and rest [−1.7 (−1.9 to −0.8) mmHg, *p* = 0.000]. There were no differences in daytime values.

**Conclusion:**

AE showed a higher PEH effect than LE sessions and rest conditions. PEH was observed in both SBP and DBP during the night. The number of studies was low, but all studies included in this meta-analysis used 24-h monitoring. The understanding of clinical relevance of AE, inducing a higher PEH, depends on a standardization of exercise protocols plus a rigorous monitoring of blood pressure.

**Systematic Review Registration:**

PROSPERO registration: CRD42021271928.

## Introduction

Recent publications in the American Heart Association (AHA) and the American College of Cardiology (ACC) recommend lifestyle approaches and physical activity as the first line of therapy for elevated blood pressure (Barone Gibbs et al., 2021). The guidelines for hypertension treatment recommend 90–150 min per week of moderate-to-vigorous intensity aerobic exercise and 90–150 min per week (6 exercises × 3 sets × 10 repetitions) of dynamic resistance exercise (Whelton et al., 2018).

An important phenomenon that occurs after exercise is post-exercise hypotension (PEH). PEH is characterized by a reduction in systolic and/or diastolic blood pressure (BP) below the control level after a single bout of exercise. PEH has been analyzed as a reduction in BP below the values immediately prior to the exercise session or in comparison with a control condition (without exercise) (Kenney and Seals, 1993). A meta-analysis that included 65 studies showed a reduction in systolic (SBP) and diastolic pressure (DBP) after exercise in pre-hypertensives (−6 mmHg) and hypertensives (−8 mmHg). Both aerobic and resistance exercises reduce SBP/DBP (6/4 mmHg) and (3/3 mmHg), respectively (Carpio-Rivera et al., 2016).

Aquatic exercise (AE) has several benefits in comparison with land exercise (LE) on account of water properties such as its density and hydrostatic pressure, which contribute to lower cardiovascular demand (Yoo et al., 2014; Chien et al., 2015) and orthopedic injury. This suggests the possibility of AE serving several individuals of different ages (Torres-Ronda and Del Alcázar, 2014).

Water-based and aquatic exercises promote several cardiovascular alterations in healthy and cardiac patients. There is a greater increase in cardiac output and pulse pressure (PP) during water immersion exercises than those performed on land. These alterations are observed at rest and during exercise (Christie et al., 1990; Gabrielsen et al., 2000; Schega et al., 2007; Schmid et al., 2007; Mourot et al., 2008). In addition, there is a reduction in the vascular tone and peripheral vascular resistance (Schega et al., 2007; Mourot et al., 2008).

A recent meta-analysis reported that AE improved vascular function, which is an important aspect of AE in reducing BP in hypertensive subjects (Igarashi et al., 2017). Considering the relevance of PEH in hypertension treatment and the differential effects of water-based/aquatic exercise on the cardiovascular system, the purpose of this meta-analysis was to evaluate whether water-based/aquatic exercise results in higher PEH than exercise on land or rest.

## Methods

This systematic review and meta-analysis followed the guidelines of the Preferred Reporting Items for Systematic Reviews and Meta-analyses (PRISMA) statement (Liberati et al., 2009). The study protocol was registered with the PRISMA statement PROSPERO (CRD42021271928).

### Research Strategy

The present systematic review was conducted using data from the following electronic databases: PubMed, Google Scholar, and EMBASE. The search terms included a combination of the following key concepts: “hypertension,” “hypotension,” “post-exercise hypotension,” “aquatic exercise,” “hypotension,” and “water-based exercise.” No language restrictions were imposed.

Two authors (C.O.T. and L.K.B.) independently conducted a systematic search. The search strategy was performed using the combination of Mesh terms and other important descriptors, as shown in the Table 1.

**Table 1:** Search strategy demonstration.

**Population**	**+**	**Independent variable**	**+**	**Dependent variable**
(“Hypertension”[Mesh] OR “High Blood Pressure”)	AND	(“Aquatic Therapy”[Mesh] OR “aquatic exercise” “water-based exercise” “water aerobics exercise” OR “Exercise”[Mesh] OR “Physical Activity” OR “Physical Activities” OR “Physical Exercise” OR “Acute Exercise” OR “Aerobic Exercise” OR “Exercise Training”)	AND	(“Hypotension”[Mesh] OR “Post-Exercise Hypotension”[Mesh] OR “Acute exercise response” OR “blood pressure response” OR “Postexercise Hypotension”)

The combination of terms defined for “population,” “independent variable,” and “dependent variable” were performed using Boolean operators. In this sense, below is an example of the command line applied to search in PubMed. (“Hypertension”[Mesh] OR “High Blood Pressure”) AND (“Aquatic Therapy”[Mesh] OR “aquatic exercise” “water-based exercise” “water aerobics exercise” OR “Exercise”[Mesh] OR “Physical Activity” OR “Physical Activities” OR “Physical Exercise” OR “Acute Exercise” OR “Aerobic Exercise” OR “Exercise Training”) AND (“Hypotension”[Mesh] OR “Post-Exercise Hypotension”[Mesh] OR “Acute exercise response” OR “blood pressure response” OR “Post-exercise Hypotension”).

### Eligibility Criteria

The criteria for the included studies were based on a checklist that considered the following characteristics: randomized control trial studies that were performed in pre-hypertensive humans (≥18 years of age), all participants were hypertensive, and the intensity and method of blood pressure measurement were described in detail. The trials compared the effect of a single session of land-based vs. water-based exercise and rest conditions, and the office and/or ambulatory BP were measured at least 15 min following the exercise bouts. The extracted data were full-text peer-reviewed. Exclusion criteria included any study that did not meet all the above inclusion criteria. The eligibility for selecting the studies was determined through the PICOS process (Table 2).

**Table 2:** Criteria for inclusion and exclusion of studies selected for review.

		**Inclusion criteria**	**Exclusion criteria**
P	Population	Hypertensive adult humans	Musculoskeletal disorders, other chronic diseases and under 18 years old
I	Intervention	A bout of water-based/aquatic exercise	Other types of exercise and chronic interventions
C	Comparison	1) Land exercise and 2) Rest control	-
O	Outcome	Blood pressure response	Blood pressure follow-up for <60 min
S	Study type	Randomized control trial	Systematic review, cross-sectional, quasi-experimental study, case reports, observational study, review, protocol study, qualitative study

### Study Selection and Quality

Two reviewers (C.O.T and L.K.B.) independently examined the titles and abstracts of all studies for eligibility. Then, the full texts of all studies that met the inclusion criteria and those in which there were some uncertainties were retrieved and reviewed by both reviewers. To reach a consensus, disagreements between reviewers were discussed with a third researcher (E.C.O). Reviewers were not blinded to the journal or authors. The rationale for deleting any full-text article was also documented.

In addition, the procedural quality of the studies was assessed using the *Tool for the assEssment of Study qualiTy and reporting in Exercise*—TESTEX (Smart et al., 2015), which is a study quality assessment and reporting tool designed specifically for use in exercise training studies. All selected studies (Terblanche and Millen, 2012; Sosner et al., 2016; Cunha et al., 2018; Júnior et al., 2019) were analyzed using the TESTEX scale. Two studies (Cunha et al., 2018; Júnior et al., 2019) scored 09, while one study (Terblanche and Millen, 2012) scored 10 and another study (Sosner et al., 2016) scored 11, which indicates high quality. These studies failed to score items 3 (allocation concealment) and 5 (supervisor blindness) concerning study quality. And in items 6a (outcome measures assessed in 85% of patients, in which more than 85% were completed), 6c (if attendance per year is reported), item 8b (if statistical comparisons between groups are reported for by minus one secondary measure) and item 10 (activity monitoring in control groups) referring to the study report. The study with score 10 (Terblanche and Millen, 2012) scored item 6a because it kept the sample at more than 85% and the study with score 11 (Sosner et al., 2016) scored item 5 because it was stated that the measure of the primary outcome evaluator was blind.

Therefore, eligible studies (Terblanche and Millen, 2012; Sosner et al., 2016; Cunha et al., 2018; Júnior et al., 2019) were classified as high quality through the quality analysis of the TESTEX scale for intervention studies. Table 3 illustrates the criteria awarded for each study.

**Table 3:** Analysis of the methodological quality of the included studies.

**References**	**Study quality**					**Partial**	**Study reporting**										**Partial**	**Total**
	**1**	**2**	**3**	**4**	**5**	**(1–5)**	**6a**	**6b**	**6c**	**7**	**8a**	**8b**	**9**	**10**	**11**	**12**	**(1–10)**	**(0–15)**
Júnior et al. (2019)	1	1	0	1	0	3	0	1	0	1	1	0	1	0	1	1	6	09
Cunha et al. (2018)	1	1	0	1	0	3	0	1	0	1	1	0	1	0	1	1	6	09
Terblanche and Millen (2012)	1	1	0	1	0	3	1	1	0	1	1	0	1	0	1	1	7	10
Sosner et al. (2016)	1	1	0	1	1	4	1	1	0	1	1	0	1	0	1	1	7	11

*Tool for the assEssment of Study quality and reporting in Exercise (TESTEX) (15) criteria: (Study quality) 1—Eligibility criteria specified; 2—Randomization specified; 3—Allocation concealment; 4—Groups similar at baseline; 5—Blinding of assessor; (Study reporting); 6—Outcome measures assessed in 85% of patients (6a = 1 point if more than 85% were completed, 6b = 1 point if adverse events were reported, 6c = if attendance to the year is reported); 7—Intention-to-treat analysis; 8—Between-group statistical comparisons reported (8a = 1 point if between-group comparisons are reported for the primary outcome variable of interest; 8b = 1 point whether statistical comparisons between groups are reported for at least one secondary measure); 9—Point measures and measures of variability for all reported outcome measures; 10—Activity monitoring in control groups; 11—Relative exercise intensity remained constant; 12—Exercise volume and energy expenditure*.

### Data Extraction

A specific data extraction file was created and used by the authors. The following study information was extracted: authors, publication year, study design, sample size, participant characteristics (sex, mean age, hypertension status), exercise protocol (intensity, duration, mode), level of water immersion (pool depth), water temperature, the method of measuring BP, and BP measurement time (Table 4).

**Table 4:** Overview of the general characteristics of the study and participants.

**References**	**Design**	**Subjects analyzed**	Age^*^ **(Yrs)**	**BP category**	**Land exercise/rest**	**Aquatic exercise**	**Pool depth**	**Water temperature**	**BP measurements (device)**	**Time point BP measurements**
Júnior et al. (2019)	Controlled clinical trial	40F	(LE-PEH) 67 ± 3 (AE-PEH) 64 ± 3 (LE) 65 ± 3 (AE) 70 ± 2	Hypertensive	Exercise session consisted of aerobic collective gymnastics (50 m: including 5 m of warm-up; 20 m of aerobic exercises at 75% of reserve heart rate; 20 m of resistance exercises; and 5m of stretching)	Exercise session consisted of a combined aerobic and resistance exercises at 75% of reserve heart rate	-	-	Heart rate monitor (POLAR^®^ RS800) and Ambulatory Blood Pressure Monitoring (ABPM)	Over 24-h post-exercise
Cunha et al. (2018)	Crossover	24F	70.0 ± 3.9	Hypertensive	The control session was a 45 m session with no exercise. During this session, participants remained seated or standing as desired. They read, talked, and drank water, but did nothing else	Exercise intensities were 55–60% of maximum heart rate (HRmax) during warm-up; 70–75% of HRmax during active exercise; and 55–60% of HRmax during cooldown.	-	-	Heart rate monitor (POLAR^®^ RS800) and ABPM	21-h post-exercise
Terblanche and Millen (2012)	Crossover	11M 10F	50 ± 12 M 54 ±10 F	Hypertensive	Exercise sessions were between 60 and 80% of peak VO_2_. Exercises included 30 m of resistance exercises followed by 25 m of aerobic exercises	Exercise sessions were between 60 and 80% of peak VO_2_ and consisted of combined aerobic and resistance exercises	The depth at the shallowest point of the pool was 2.1 meters	27°C	Automated ambulatory air bladder-containing cuff (Ergoline Ergoscan 2008, Germany)	24-h post-exercise
Sosner et al. (2016)	Parallel	22M 20F	(MICE) 65 ± 6 (HIIE-D) 65 ± 8 (HIIE-I) 63 ± 9	Hypertensive	On an electromagnetically braked cycle ergometer, each exercise session was preceded by a 5 m of warm-up consisting in pedaling at 60 W with a cadence of 80 revolutions per min (rpm) and followed by a 5 m of recovery period in a sitting position that began immediately after exercise cessation. The intensity was determinate based in Maximal Continuous Graded Exercise Test potency	On a mechanically braked cycle ergometer, each exercise session was preceded by a 5 m of warm-up consisting in pedaling at 40 rpm and followed by a 5 m of passive recovery period in a sitting position that began immediately after exercise cessation. The intensity was determinate based in Maximal Continuous Graded Exercise Test potency	Saddle and handlebar height as well as forward placement were adjusted to determine optimal position. Immersed cycling (up-to-the-chest)	30°C	Ambulatory BP (Mobil-O-Graph PWA)	24-h post-exercise

*ABPM, Ambulatory Blood Pressure Monitoring; BP, Blood Pressure; HRmax, maximum heart rate; M, male; F, female; W, watts; RPM, revolutions per minute; m, minutes; s, seconds;*

* *Mean ± SD; Yrs, years; AE-PEH, aquatic exercise post-exercise hypotension; LE-PEH, land exercise post-exercise hypotension; AE, aquatic exercise; LE, land exercise; MICE, moderate-intensity continuous exercise; HIIE-D, high-intensity interval exercise dryland; HIIE-I, high-intensity interval exercise immersed*.

### Statistical Analyses

Analyses were performed using Comprehensive Meta-Analysis software (CMA, version 2.2.064, Biostat, NJ, USA). Two-sided statistical significance was set at *p* < 0.05. The primary outcome measure was an effect on blood pressure response. Descriptive data of treatment groups and participants are reported as the mean ± SD. Study data were pooled using a random-effects model. Inconsistencies were estimated using the *I*^2^ statistic. Additionally, we evaluated an additional hypothesis that there might be differences in the effects of AE on post-exercise hypotension accordingly to the comparison type (land exercise or rest control). Differences between subgroups were analyzed by means of an analysis of variance (*Q*-test-based ANOVA). Additionally, the Duval and Tweedie (2000) trim and fill computation were used to estimate the effect of publication bias on the results.

## Results

**Figure 1:**
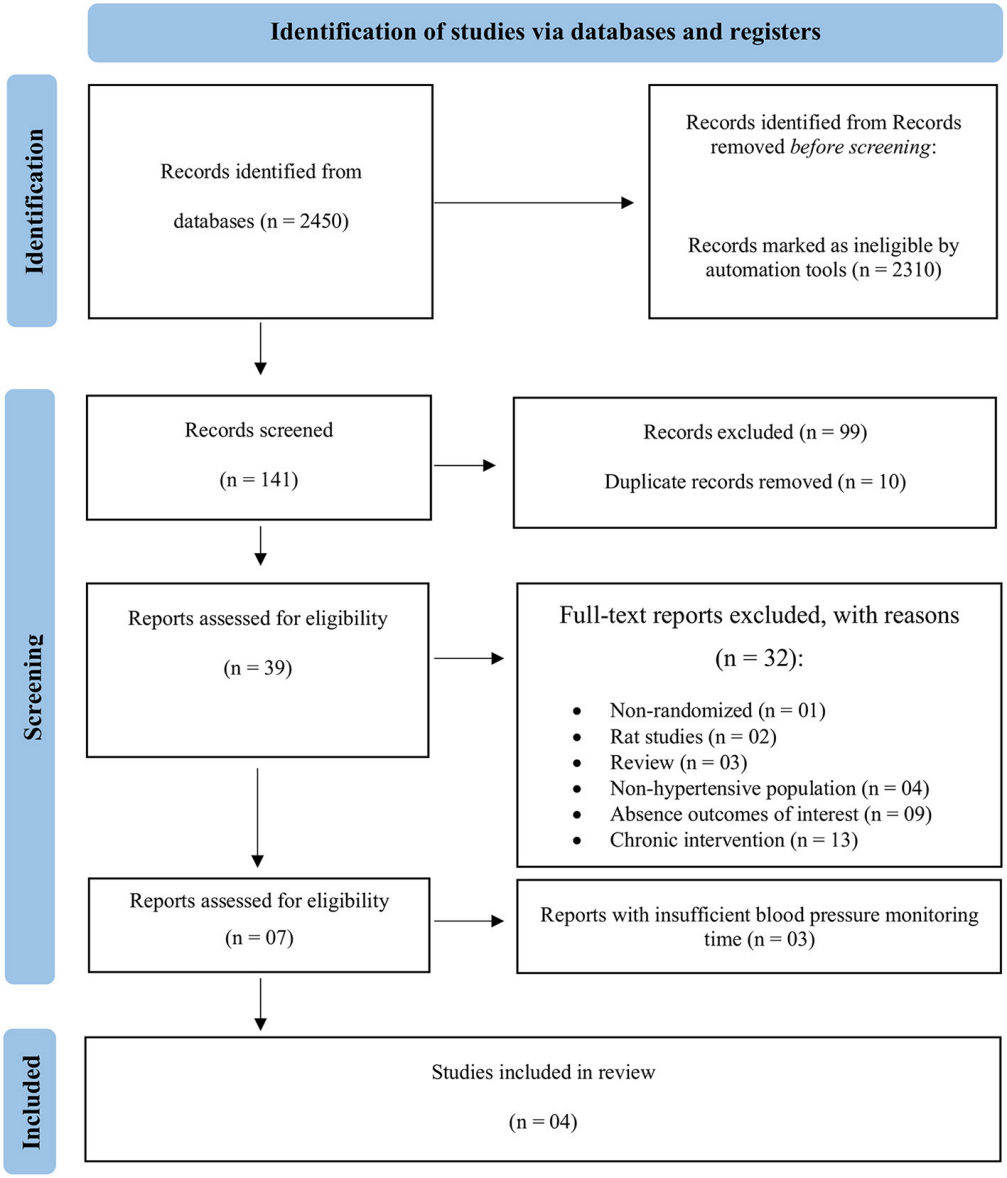
PRISMA flow diagram showing the selection process of eligible articles.

A PRISMA flow diagram of the literature search and selection process is shown in Figure 1. The priori search identified 2,450 articles involving PEH. After the screening process, 141 abstracts were read, 99 articles were excluded for duplicates or other reasons, 39 articles were selected as potential studies for evaluation, 32 full-text reports were excluded, seven reports were assessed for eligibility, and after analyses, three reports were excluded due to insufficient blood pressure monitoring time. Only four articles were selected, finally (Terblanche and Millen, 2012; Sosner et al., 2016; Cunha et al., 2018; Júnior et al., 2019). These studies were included in the final meta-analysis because they were conducted in hypertensive volunteers and because PEH was recorded 24-h after aquatic, land, or rest conditions.

### Risk of Bias Within and Across Studies

The TESTEX scale uses 12 criteria, with some criteria scoring more than one possible point, for a maximum score of 15 points. The overall quality of the included studies was of high quality, all studies scored above 09. Weaknesses in the studies were: allocation concealment, whether attendance per year is reported, whether statistical comparisons between groups are reported for at least one secondary measure, and activity monitoring in control groups (Terblanche and Millen, 2012; Sosner et al., 2016; Cunha et al., 2018; Júnior et al., 2019).

The kappa correlation showed high overall agreement between the researchers [*k* = 0.93; 0.73–1.00 (95% CI)—*p* < 0.001].

The Duval and Tweedie (2000) correction model was applied to the AE study groups for PEH. No trimmed studies could be identified. The consistency of studies was analyzed using *I*^2^ as a test of heterogeneity for subgroup analyses (Table 5).

**Table 5:** Heterogeneity of studies.

	**Heterogeneity test**			
**Control**	***n***	***Q***	***p***	***I* ^2^**
**Systolic blood pressure–24 h**				
Land exercise	3	32.9	0.000	93.9
Rest	3	1.7	0.413	0.0
Overall	6	34.7	0.000	85.6
**Systolic blood pressure—daytime**				
Land exercise	3	63.0	0.000	96.8
Rest	3	0.7	0.671	0.0
Overall	6	156.7	0.000	96.8
**Systolic blood pressure—nighttime**				
Land exercise	3	17.5	0.000	88.6
Rest	3	1.0	0.599	0.0
Overall	6	78.7	0.000	93.6
**Diastolic blood pressure–24 h**				
Land exercise	3	18.0	0.000	88.9
Rest	3	0.6	0.713	0.0
Overall	6	20.3	0.001	75.4
**Diastolic blood pressure—daytime**				
Land exercise	3	28.7	0.000	93.0
Rest	3	1.1	0.569	0.0
Overall	6	81,0	0.000	93.8
**Diastolic blood pressure—nighttime**				
Land exercise	3	2.6	0.269	23.8
Rest	3	0.6	0.728	0.0
Overall	6	22.4	0.000	77.7

*N, number of trials; Q, observed variation; I^2^, ratio of true heterogeneity to the total variation in observed effects*.

### Study Characteristics

Table 4 shows the characteristics of these studies. The characteristics of the sample included (Terblanche and Millen, 2012; Sosner et al., 2016; Cunha et al., 2018; Júnior et al., 2019) in the trials were as follows: 33 men and 94 women, a water group (*n* = 46) (Sosner et al., 2016; Cunha et al., 2018; Júnior et al., 2019), land group (*n* = 34) (Sosner et al., 2016; Júnior et al., 2019), and a rest or control group (*n* = 12) (Cunha et al., 2018); one study (Terblanche and Millen, 2012) (*n* = 21) in which the participants performed one land and one water exercise session in random order, as well as a control session with no exercise. Two studies (Terblanche and Millen, 2012; Cunha et al., 2018) were crossover-type studies, one study (Júnior et al., 2019) was a controlled clinical trial, and the other (Sosner et al., 2016) was a parallel study. In one study (Sosner et al., 2016), the volunteers were allocated to two groups: high-intensity dry land or high intensity in immersed condition, and in another (Terblanche and Millen, 2012; Cunha et al., 2018; Júnior et al., 2019), the volunteers were allocated to aquatic and land groups using a stratified randomization method. The mean ages of the participants in the studies were 53–65 years. All studies (Terblanche and Millen, 2012; Sosner et al., 2016; Cunha et al., 2018; Júnior et al., 2019) reported that the subjects were hypertensive; only one study included high blood pressure (systolic ≥ 130 mmHg and diastolic ≥ 85 mmHg) and hypertensive subjects. Exercise intensity was expressed as heart rate reserve (75%) (Júnior et al., 2019). One study (Sosner et al., 2016) expressed exercise intensity using the percentage of maximal grade test (60–80%), and one study (Terblanche and Millen, 2012) used the VO_2_ maximal percentage (60–80%). Only two studies (Terblanche and Millen, 2012; Sosner et al., 2016) reported the depth of water and temperature. Four studies (Terblanche and Millen, 2012; Sosner et al., 2016; Cunha et al., 2018; Júnior et al., 2019) used automatic ambulatory BP monitors for 24 h. Supplementary Material shows the office SBP and DPB and more details of exercise prescription parameters.

### Main Outcomes

#### Systolic Blood Pressure

**Figure 2:**
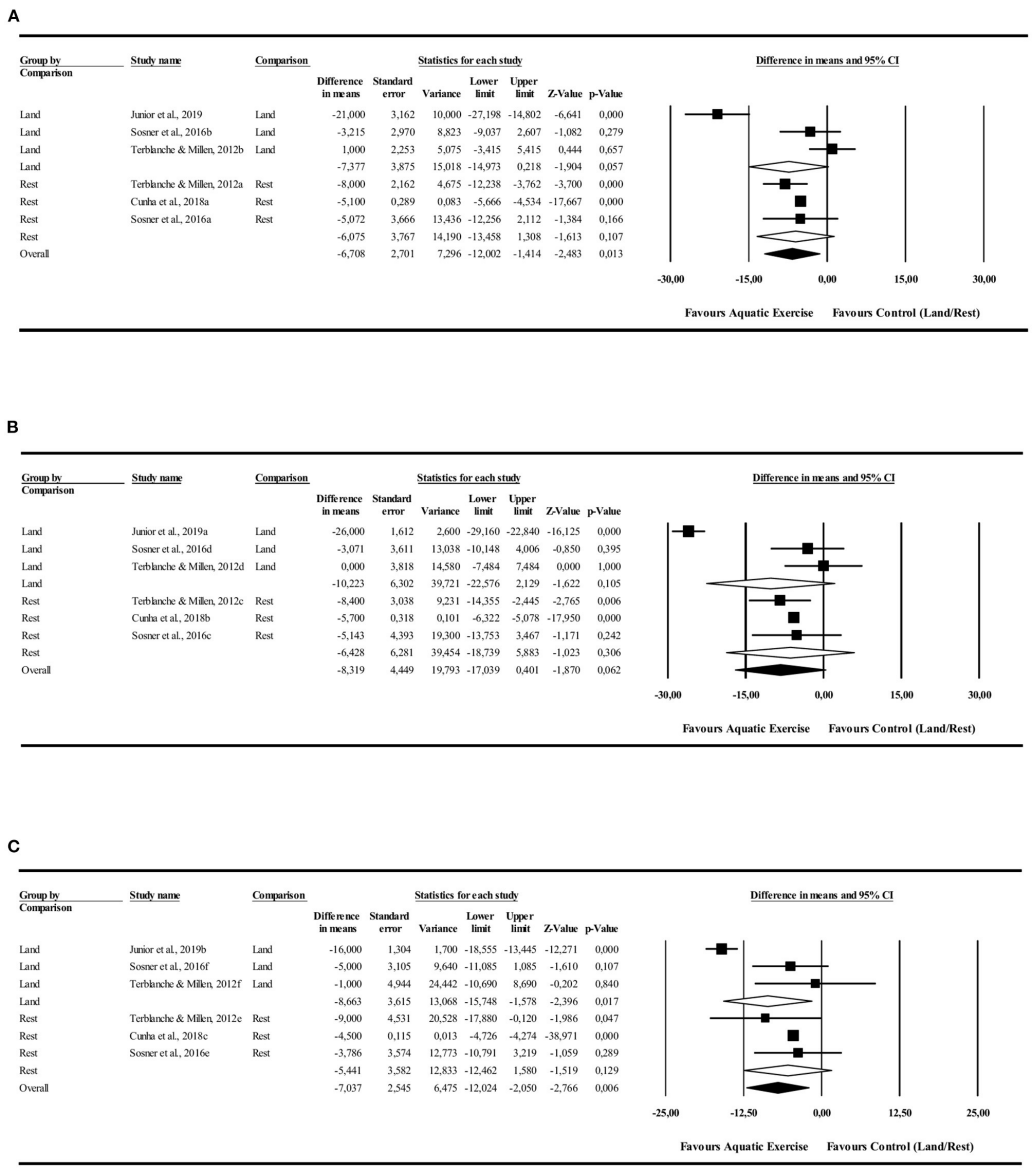
Systolic blood pressure post-exercise hypotension effect. Comparison between water exercise (aquatic) with land exercise or rest condition. **(A)** 24 h, **(B)** Daytime, **(C)** Nighttime.

The 24-h analysis did not identify PEH for AE compared to LE [−7.3 mmHg (−14.9 to −0.2 mmHg), *I*^2^ = 93%, *p* = 0.057] or rest [−6.0 mmHg (−12.0 to −1.4 mmHg), *I*^2^ = 0%, *p* = 0.1] conditions. Monitoring during the night showed a significant PEH for AE compared to LE [−8.6 mmHg (−15.0 to −1.5 mmHg), *I*^2^ = 88%, *p* = 0.01] and no significant favorable effect in relation to control rest [−5.4 mmHg (−12.8 to 1.5 mmHg), *I*^2^ = 0%, *p* = 0.12]. Daytime analysis did not identify significant PEH in favor to AE compared to LE [−10.2 mmHg (−22.5 to 2.1 mmHg), *I*^2^ = 96%, *p* = 0.105] or control rest [−6.4 mmHg (−18.7 to 5.8 mmHg), *I*^2^ = 0%, *p* = 0.306] (Figure 2).

#### Diastolic Blood Pressure

**Figure 3:**
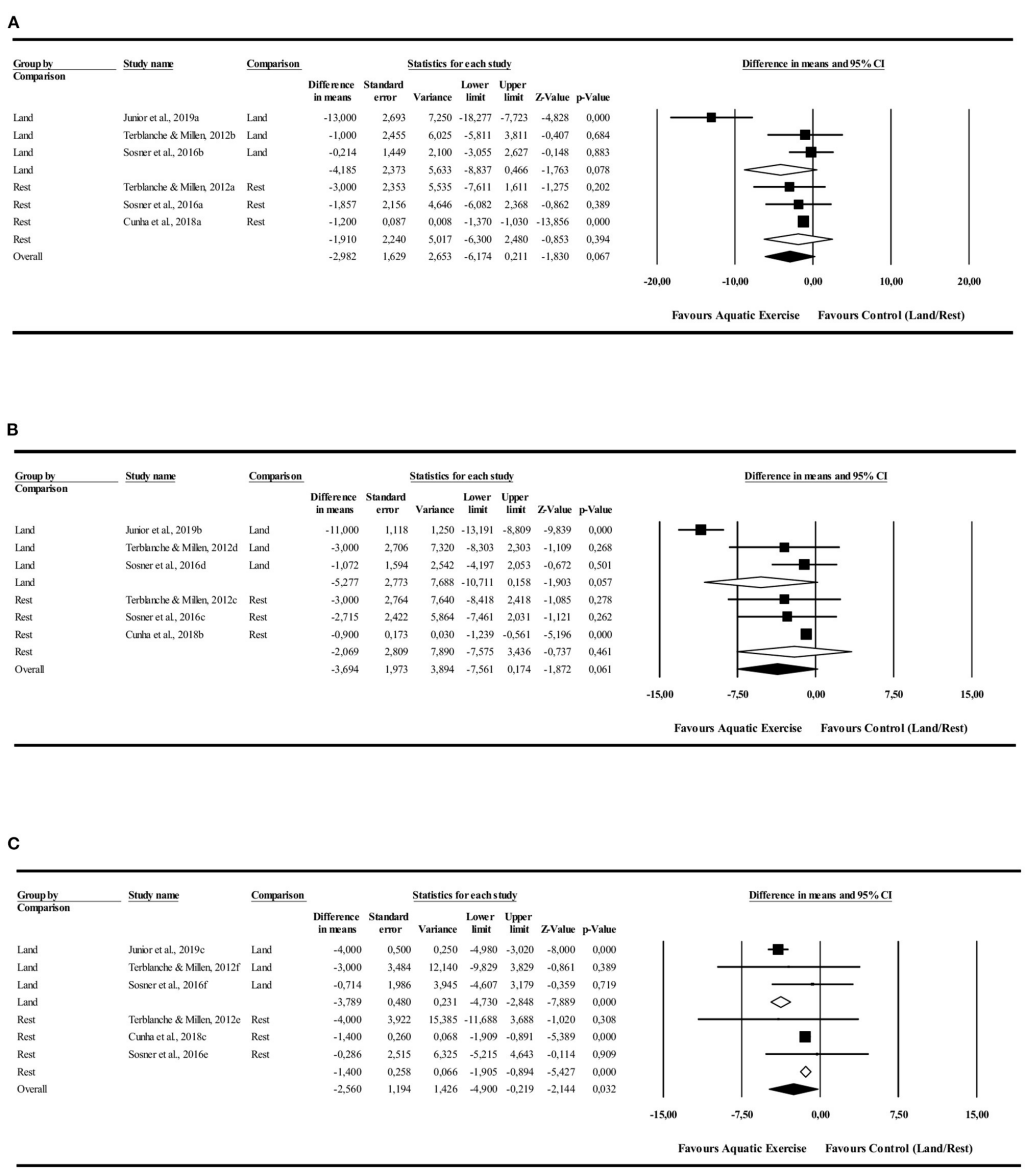
Diastolic blood pressure post-exercise hypotension effect. Comparison between water exercise (aquatic) with land exercise or rest condition. **(A)** 24 h, **(B)** Daytime, **(C)** Nighttime.

The 24-h analysis did not identify differences in favor to AE in comparison to LE [−4.1 mmHg (−8.8 to 0.4 mmHg), *I*^2^ = 88%, *p* = 0.07] or rest [−1.9 mmHg (−6.0 to −2.4 mmHg), *I*^2^ = 0%, *p* = 0.06] control conditions. The AE had favorable PEH during the night in comparison with LE [−3.7 mmHg (−4.7 to −2.8 mmHg), *I*^2^ = 23%, *p* = 0.000] and rest [−1.7 mmHg (−1.9 to −0.8 mmHg), *I*^2^ = 0%, *p* = 0.000] control conditions. There were no differences in the daytime values in comparison to LE [−5.2 mmHg (−10.7 to 0.1 mmHg), *I*^2^ = 93%, *p* = 0.057] and rest [−2.0 mmHg (−7.5 to 3.4 mmHg), *I*^2^ = 0%, *p* = 0.461] control conditions (Figure 3).

The mean effects were similar for “control conditions” (LE vs. rest), except for DBP during the nighttime period of blood pressure monitoring, where the magnitude of blood pressure reduction was greater in relation to LE (*p* = 0.000; *Q*-test-based ANOVA).

## Discussion

The purpose of the present systematic review and meta-analysis was to determine whether AE induces significant PEH compared to LE and rest conditions. The pooled results suggest that a single session of AE induced a statistically significant PEH during the night for both SBP and DPB; for DBP, the AE led to different results between land and rest.

An interesting recent review (Zhou et al., 2021) examined AE for health promotion during a 31-year bibliometric analysis. The results suggest that research on this topic has been constantly increasing over the past 30 years, and trends have focused on improving cardiovascular health with AE.

One meta-analysis that investigated the chronic effects of AE on BP showed a significant decrease in BP; the SBP was estimated to be −8.4 mmHg while the change in DBP was estimated to be −3.3 mmHg (Igarashi and Nogami, 2017). These reductions are higher than those observed in other exercise modalities: regular endurance exercise led to a decrease of approximately −3.5 mmHg (SBP) and −1.8 mmHg (DBP) (Cornelissen and Smart, 2013); resistance exercises led to a decrease of −3.9 mmHg (SBP) and −3.6 mmHg (DBP) (Cornelissen et al., 2011); and yoga exercise training led to a decrease of −5.2 mmHg (SBP) and −5.0 mmHg (DBP) (Chu et al., 2014).

The mechanisms by which aquatic/water-based exercise reduces BP must be further investigated. Currently, research shows that these exercises result in a reduction in peripheral vascular resistance (Pendergast et al., 2015), suppression of the renin-angiotensin system, and cardiopulmonary and baroreflex activation (Gabrielsen et al., 1996; Reilly et al., 2003) that lead to a marked reduction in renal sympathetic nerve activity and increased urine flow and sodium excretion (Larochelle et al., 1994). These alterations are mediated by hydrostatic effects of immersion in water, which redirects ~700 mL of blood flow from the extremities (increased venous return) to the thorax (Sik Park et al., 1999; Meredith-Jones et al., 2011).

Another important point that must be observed in further works is the standardization of participants' physical activity level. Only two studies included in this review describe the physical level of voluntaries. In one of them (Júnior et al., 2019), the hypertensive subjects were trained in AE or land exercise at least for 6-months before the experiment. Another study described that the voluntaries were trained only in land (Cunha et al., 2018). The forest plot showed that the AE trained group (6-months) had higher PEH than the land trained group. However, both groups of hypertensive subjects showed significant PEH in favor of AE compared to land or rest. On the other hand, in two articles (Terblanche and Millen, 2012; Sosner et al., 2016) that do not describe if the participants were physically active, AE was not better, with an exception for SBP (Terblanche and Millen, 2012). These results can indicate that participants' physical activity level can impact PEH.

The pooled net change contained significant heterogeneity, but the reason for this could not be determined. Probably the active level of participants can be one reason. Physically active individuals achieved higher PEH after the exercise session. This seems to support the theory proposed by some authors (Hamer, 2006). Some physiological mechanisms produced by the chronic effect of exercise that reduce BP also play a role in PEH onset. For example, exercise training has been shown to lead to important adaptations and better arterial vessel compliance that may facilitate the decrease in peripheral resistance following an exercise session (Thijssen et al., 2013). It has been also demonstrated that trained hypertensive patients presented lower values of SBP and DBP than the sedentary participants after a single bout of continuous aerobic exercise (Imazu et al., 2017).

The adaptations to AE can collaborate with PEH. Training seems to generate neurohumoral adaptations that are important for BP control, and chronic AE (12-weeks) in resistant hypertensive subjects showed a significant decrease in 24-h SBD and DBP in the clinic; concomitantly, nitric oxide levels increased, and endothelin-1, renin, and norepinephrine levels showed significant reductions (de Cruz et al., 2017). The confirmation of these effects must justify the further clinical use of AE training for treatment of hypertensive subjects.

Another important question is the modality and/or way the exercise was performed. Three studies included in this meta-analysis used a combination of aerobic and resistance exercise in water or land; one used only aerobic exercise, and the last used High Intensive Interval Exercise (HIIE). A recent meta-analysis showed that land HIIE promoted a larger PEH than moderate-intensity continuous exercise on ambulatory daytime blood pressure (Marçal et al., 2021). Another meta-analysis showed that several types of regular AE as swimming, deep water, circuits, resistance, and others, significantly reduce the blood pressure in hypertensive subjects. The pooled net results of the present work showed significant PEH, especially during the nighttime, so it seems that the AE, independently of how it is done, leads to significant PEH (Igarashi and Nogami, 2017) characterizes one more area for future studies.

One study (Júnior et al., 2019) included in this meta-analysis shows that PEH is higher for the AE group only in the 12th hour after exercise session compared to the land group. This point of ambulatory blood pressure measurement is characterized as nighttime evaluation. Another study (Cunha et al., 2018) finds more points of differences between AE and rest for DBP in 4th, 11th, 12th, and 13th h after exercise session, showing the predominance of differences in nighttime period. The exact underlying mechanisms by which exercise improves nighttime PEH are not clear. One possible mechanism involved can be the effects of AE in autonomic control. The blood pressure and heart rate are modulating by autonomous nervous system, which exhibits a predominant vagal tone in most species, including humans. Bocalini et al. (2017) measured the heart rate variability (HRV) which indicated the predominance of sympathetic (Low Frequency—LF) and vagal modulation (High Frequency—HF), 90-min after land and AE session. After both land and AE, the HF was significantly greater than that assessed at rest condition, suggesting that more than just a parasympathetic reactivation in PEH occurs. In addition, the increase in HF and the reduction in LF and LF/HF ratio during recovery were also significantly greater in the AE than in the land-based exercise, reinforcing the effectiveness of exercise under immersion, especially for hypertensive patients. During nighttime there is a prevalence of vagal tone (Furlan et al., 1990), the AE can contribute to increased vagal modulation by alterations in the ratio of sympathetic/vagal tone once the decrease in sympathetic activity is a mechanism described for AE (Schmid et al., 2007).

There are very few studies that have compared the chronic effects of AE and LE on blood pressure (BP) control. Júnior et al. (2019) observed that elderly hypertensive individuals trained in an aquatic setting had lower baseline BP during the daytime; Arca et al. (2013) and Ruangthai et al. (2020) showed the same effect for BP, but a study in patients with peripheral artery disease revealed that although land-based exercise therapy is effective in reducing arterial stiffness, heated-water exercise demonstrates greater benefits on vascular function (Park et al., 2020).

Interestingly, recent data (Ruangthai et al., 2020) showed a better lipid profile for AE compared to LE, suggesting that the improvement in blood lipid levels and body fat after aquatic training programs might result from the influence of water temperature. Studies have shown a positive effect of AE on blood lipids and body composition (Takeshima et al., 2002), temperatures of 28–30°C for 60 min, indicating that temperature can influence metabolic rate. This response is due to the increased activity of the sympathetic nervous system (Daanen and Van Marken Lichtenbelt, 2016).

A study examining patients with stable chronic heart failure showed that AE has additional benefits to endothelial function because this type of exercise effectively increases the basal level of plasma nitrates (Mourot et al., 2009). Water immersion decreases the vascular tone and total peripheral resistance (Mourot et al., 2008). A study conducted on a dog model showed an increase in skeletal muscle blood flow in the forelimbs and hindlimbs during immersion, suggesting an increased peripheral blood flow (Hajduczok et al., 1987). The aquatic immersion effect led to increased shear stress on vessel walls, increasing nitric oxide bioavailability (Niebauer and Cooke, 1996; Green et al., 2004). Thus, water/aquatic-based exercises may have different effects on vessel responses compared to LE, and further studies are required to investigate these effects.

The practical clinical importance of AE is the possibility of adherence to an exercise program. The water proprieties enable the participation of several people, for example, elderly subjects, patients who cannot support their weight, or have poor balance. Another important point with great clinical relevance is a cardiovascular response in AE, the SBP, DBP, and HR gradually increased during the underwater treadmill walking, but their mean maximum increases were significantly smaller than those of the land treadmill walking, underwater treadmill walking can better help relieve the cardiovascular workload compared to the land treadmill walking in stroke patients (Yoo et al., 2014). Additionally, AE allows the combination of aerobic and resistance exercises due to the buoyancy effect, which may potentiate the effects of exercise as well-known effects of exercise, such as autonomic activity modulation, better baroreceptor reflex sensitivity, and endothelium-dependent vasodilatation (Fadel, 2008).

The clinical relevance of AE in PEH must be more evident after the elucidation of magnitude and duration time of the PEH, so, at the end of this discussion, some future perspectives will be indicated. In these meta-analyses was evident that AMBP monitoring is scarce. The use of ABPM has limitations due to the adherence of the equipment in arms for 24-h. In addition, the equipment is expensive. One alternative is the home blood pressure monitoring, at least 2 or 3-times at nighttime. Another important question is the standardization of participants' activity level, as well the monitoring of the daily life activities during the intervention period of the experiment. Investigators can use a step counter or another device for these monitoring. Another gap that needs investigation is the autonomic control during and after AE. In these perspectives, a simple and applicable methodology of measuring heart rate variability can be used. Baroreflex sensibility is another parameter that demands more investigation, considering the importance of neural mechanisms involved in blood pressure control. This measurement presents more limitations, but several no invasive methods can be applied: Valsalva maneuver, which produces a natural challenge for the baroreceptors by voluntarily increasing intrathoracic and abdominal pressure through straining; the neck chamber technique, which allows a selective activation/deactivation of carotid baroreceptors by application of negative/positive pressure to the neck region and spontaneous oscillations of systolic arterial pressure and RR interval.

## Conclusion

AE is effective in promoting PEH during the night for SBP in comparison with LE and for SBP and DPB in comparison with the rest condition. The 24-h analysis did not show any significant differences. The number of studies describing a 24-h measure was low, and further studies are required, including different physical conditions (active or sedentary) and the type of exercise enrolled (aquatic or land). Additionally, the nighttime results draw attention to possible aquatic and water-based effects in autonomic control of BP. The benefits provided by this type of exercise warrant for this research topic to be further explored.

## limitations

The current meta-analysis has several limitations. The pooled net change for land comparisons contained significant heterogeneity, but the reason for this heterogeneity could not be determined. Several important questions need to be addressed: the physical level of volunteers was described in only two papers, and in one of them (Cunha et al., 2018) the subjects were physically active on land but not in water; in another study (Júnior et al., 2019), the study population consisted of elderly hypertensive individuals, of which 20 trained in land-based exercises and 20 in aquatic-based exercises. The participants were enrolled in recurrent physical exercise for at least 6 months before evaluation for a minimum of two sessions a week.

Another important point that can contribute to high heterogeneity to land exercise is the standardization of exercise session between studies, one study was made in cycle ergometer (Sosner et al., 2016) and other 3 studies made through dynamic whole-body exercise (Terblanche and Millen, 2012; Cunha et al., 2018; Júnior et al., 2019), in addition, the intensity of exercise is controlled by different methods: HR reserve, HR maximal, VO_2_ maximal and RPM. More studies with similar protocols are necessary to evaluate the aquatic vs. land exercise effect in blood pressure.

## Data Availability Statement

The raw data supporting the conclusions of this article will be made available by the authors, without undue reservation.

## Author Contributions

CT, LB, JC, and EO contributed to the conception and design of the manuscript. CT and LB performed the data search and data extraction. JC performed the data analysis. CT, EO, and DC drafted the manuscript. JC, LB, and DC performed critical revisions of the manuscript. All authors contributed to the manuscript and approved the submitted version.

## Funding

This study was funded by National Council for Scientific and Technological Development (Conselho Nacional de Desenvolvimento Científico e Tecnológico—CNPq), Coordination and Improvement of Superior Education Staff (Coordenação de Aperfeiçoamento de Pessoal de Nível Superior—CAPES), Minas Gerais State Research Support Foundation (Fundação de Amparo à Pesquisa do Estado de Minas Gerais—FAPEMIG), and Pro-Rectory of Research, Postgraduate and Innovation of the Federal University of Ouro Preto (Pró-Reitoria de Pesquisa, Pós-Graduação e Inovação da UFOP—PROPPI-UFOP).

## Conflict of Interest

The authors declare that the research was conducted in the absence of any commercial or financial relationships that could be construed as a potential conflict of interest.

## Publisher's Note

All claims expressed in this article are solely those of the authors and do not necessarily represent those of their affiliated organizations, or those of the publisher, the editors and the reviewers. Any product that may be evaluated in this article, or claim that may be made by its manufacturer, is not guaranteed or endorsed by the publisher.
