# Supplementary material for: Effects of Aquatic Exercise in Post-exercise Hypotension: A Systematic Review and Meta-Analysis
Source: Front Physiol. 2022 Jan 31;13:834812. doi: 10.3389/fphys.2022.834812 (PMC8841763; doi:10.3389/fphys.2022.834812)
Supplement: Supplementary file 1 [file Table_1.DOCX]

Supplementary Material

# Supplementary File 1

|  | | | | | |
| --- | --- | --- | --- | --- | --- |
| **Supplementary File 1**\| Complementary data acquired from eligible studies | | | | | |
| **Study** | **Office SBP/DBP (mmHg)** | **Exercise modality** | **Ergometer/exercise execution** | **Land exercise/REST** | **Water Exercise** |
| Júnior et al. (2019) | 140 ± 4.4/ 85 ± 4.3 | Combined: Continuous aerobic exercise plus strength session 75% of reserve HR (RHR) | Whole body | Aerobic collective gymnastics with a duration of 50 minutes, including five minutes of preparatory activity, 20 minutes of aerobic exercises at 75% of RHR, 20 minutes of resistance exercises, and five minutes of stretching. | Aerobic collective gymnastics with duration of 50 minutes, comprising five minutes of preparatory activity, 20 minutes of aerobic exercises at 75% of reserve HR (RHR), 20 minutes of strength exercises, and five minutes of stretching. |
| Cunha et al. (2018) | 124.0 ± 13.0/ 72.3 ± 8.3 | Continuous aerobic exercise 70–75% HRmax | Whole body | Rest by 45- minute with no exercise. During this session, participants remained seated or standing as desired. They read, talked, and drank water, but did nothing else. | Dynamic warm-up period (5 minutes), an active exercise period (35 minutes), and a cooldown period (5 minutes) The training session consisted of 18 groups of exercise performed continuously, each lasting on average 2 minutes and 30 seconds. Of these groups, 4 were lower limb training exercise; 4 upper-limb; and 10 both upper- and lower-limb (combined). Each session had 3 components: warm-up including 2 combined exercise groups; cooldown at the end of the session; and the main part including 4 upper-limb, 4 lower-limb, and 6 combined exercise groups, alternating body segments. |
| Sosner et al. (2016) | 143.1 ±13.8/85.1± 9.2 | High-Intensity Interval Exercise Sessions (20 minutes) Sets of 10 minutes composed of repeated phases of 15 seconds at 100% of peak power output interspersed by 15 seconds of passive recovery. Four minutes of passive recovery were allowed between the two sets, and a 5-minute cooldown after the last 15- second exercise phase, immediately followed by a 5-minute period of passive recovery in a seated position. | Cycle | Each exercise session was preceded by a 5- minute warmup consisting in pedaling at 60 W with a cadence of 80 rounds per minute (rpm), and followed by a 5-minute recovery period in a sitting position that began immediately after exercise cessation. | External power output was determined from pedaling cadence (in rpm). Each exercise session was preceded by a 5-minute warm-up consisting in pedaling at 40 rpm, and followed by a 5- minute passive recovery period in a sitting position that began immediately after exercise cessation. |
| Terblanche and Miller (2012) | 145 ± 13/ 94 ± 12 | Combined: Continuous aerobic exercise plus strength Continuous exercise between 60- 80% of peak VO2 | Whole body | The land exercise session consisted of combined aerobic and strength exercises. The aerobic exercises included 10 min each of treadmill walking, skiing on an elliptical trainer and cycling on a stationary bike. The resistance exercises followed the aerobic exercises and lasted 25 min. This included incline leg press, seated leg curl, leg extension, bench press, shoulder press, latissimus dorsi pulldown, seated row, triceps extension and biceps curl. Two sets of 10 repetitions were performed, with a rest period of 30 s between each set and 90 s between each exercise. | Exercises included 30 min of endurance type activities, i.e. water treading, walking and jogging with and without rhythmic arm movements below and slightly above the water level. These activities were alternated with resistance type exercise, i.e. arm and leg movements against the resistance of the water with the body in the vertical position for a total of 25 min. |
|  | | | | | |
